# Electrospun SF/PLCL nanofibrous membrane: a potential scaffold for retinal progenitor cell proliferation and differentiation

**DOI:** 10.1038/srep14326

**Published:** 2015-09-23

**Authors:** Dandan Zhang, Ni Ni, Junzhao Chen, Qinke Yao, Bingqiao Shen, Yi Zhang, Mengyu Zhu, Zi Wang, Jing Ruan, Jing Wang, Xiumei Mo, Wodong Shi, Jing Ji, Xianqun Fan, Ping Gu

**Affiliations:** 1 Department of Ophthalmology, Ninth People's Hospital, Shanghai Jiao Tong University School of Medicine, Shanghai, 200011, P.R. China; 2 Biomaterials and Tissue Engineering Laboratory, College of Chemistry & Chemical Engineering and Biotechnology, Donghua University, Shanghai, 201620, P. R. China

## Abstract

Biocompatible polymer scaffolds are promising as potential carriers for the delivery of retinal progenitor cells (RPCs) in cell replacement therapy for the repair of damaged or diseased retinas. The primary goal of the present study was to investigate the effects of blended electrospun nanofibrous membranes of silk fibroin (SF) and poly(L-lactic acid-co-ε-caprolactone) (PLCL), a novel scaffold, on the biological behaviour of RPCs *in vitro*. To assess the cell-scaffold interaction, RPCs were cultured on SF/PLCL scaffolds for indicated durations. Our data revealed that all the SF/PLCL scaffolds were thoroughly cytocompatible, and the SF:PLCL (1:1) scaffolds yielded the best RPC growth. The *in vitro* proliferation assays showed that RPCs proliferated more quickly on the SF:PLCL (1:1) than on the other scaffolds and the control. Quantitative polymerase chain reaction (qPCR) and immunocytochemistry analyses demonstrated that RPCs grown on the SF:PLCL (1:1) scaffolds preferentially differentiated toward retinal neurons, including, most interestingly, photoreceptors. In summary, we demonstrated that the SF:PLCL (1:1) scaffolds can not only markedly promote RPC proliferation with cytocompatibility for RPC growth but also robustly enhance RPCs’ differentiation toward specific retinal neurons of interest *in vitro*, suggesting that SF:PLCL (1:1) scaffolds may have potential applications in retinal cell replacement therapy in the future.

Retinal degenerative diseases, including retinitis pigmentosa and age-related macular degeneration, seriously threaten human health^1^. Many solutions have been proposed, including photosensitive chip transplantation, gene therapy, antiangiogenic therapy, growth factor additives and cell transplantation therapy. Retinal progenitor cells (RPCs) have been a focus of transplantation therapies since Klassen HJ and co-workers isolated RPCs and demonstrated that they not only are capable of differentiating into retinal neurons but also possess integrative abilities similar to those of brain-derived stem or progenitor cells^2^,^3^,^4^. RPCs can currently be successfully isolated and cultured *in vitro* and maintain their ability to differentiate into both neuronal and glial lineages^5^. However, scientists are concerned by the limited capacity of RPCs to expand and differentiate into retinal neurons, including photoreceptors^6^,^7^. Many efforts have been made to extend this capacity, including improvements in the isolation methods, changes to the culture media and the application of a culturing carrier^8^,^9^,^10^,^11^,^12^,^13^. Previous studies have demonstrated that substrates, such as PCL, PCL with laminin, and PCL with chitosan electrospun nanofibres, can enhance cell attachment, proliferation or differentiation and promote the expression of genes specific to photoreceptor cells or bipolar cells^14^,^15^,^16^. The second problem for the future clinical application of RPCs is how to effectively deliver RPCs to the retina and ensure their ability to integrate into the retina and differentiate into retinal neuronal cells. In general, the direct injection of a cell suspension using a needle leads to poor cell survival and migration due to the shearing forces induced during cell injection and reflux^17^. By contrast, biodegradable polymer scaffolds can deliver these cells to the subretinal space in a more organised manner than bolus injections and would providing a laminar organisation and structural guidance channels to the graft. The scaffold delivery strategy has been shown to enhance cell survival and direct cell differentiation in a variety of retinal degenerative models^18^,^19^,^20^,^21^.

Electrospinning is an fabrication technique capable of producing fibres ranging from a few nanometres to hundreds of microns and has been used to produce nanofibrous scaffolds that can generate interconnected porous nanofibrous scaffolds with higher porosity, allowing an exchange of nutrients and a higher surface area and thereby mimicking the topographic features of the extracellular matrix (ECM)^22^,^23^. Electrospun poly(L-lactic acid-co-ε-caprolactone) (PLCL) scaffolds are a copolymer of L-lactic acid and e-caprolactone whose mechanical properties and degradation rate can be controlled by changing the L-lactic acid/ε-caprolactone molar ratios^24^. Electrospun PLCL nanofibres have been demonstrated to support the growth and proliferation of many cell types while showing inadequate cell affinity due to the absence of recognition sites for cell adhesion. Silk fibroin (SF) has been widely used in tissue engineering for artificial ligaments, blood vessels, bone, and nerves because of its obviously unique properties, including good biocompatibility, good oxygen and water vapour permeability, a wide range of molecular structures, slow degradability, low inflammatory response and controllable morphology^25^,^26^. The blending of bioactive SF with the beneficial mechanical properties of PLCL to produce a new biohybrid material may support RPC growth.

In this study, we investigated the effects of electrospun interconnected and porous nanofibrous scaffolds composed of SF and PLCL on retinal progenitor growth. The primary objective of the present study was to evaluate the proliferative capability and differentiation potential of RPCs seeded on SF/PLCL scaffolds *in vitro*. Our data demonstrate that different concentrations of SF/PLCL nanofibrous scaffolds performed well but showed different bioactivities for RPC growth. In particular, the data obtained with the SF:PLCL (1:1) scaffolds demonstrate that these can not only enhance RPC proliferation but also promote RPC differentiation toward retinal neurons, such as rhodopsin-positive photoreceptor cells, indicating that electrospun SF:PLCL (1:1) scaffolds may be useful in retinal cell replacement therapies.

## Results

### Morphology of electrospun SF/PLCL nanofibrous scaffolds

**Figure 1:**
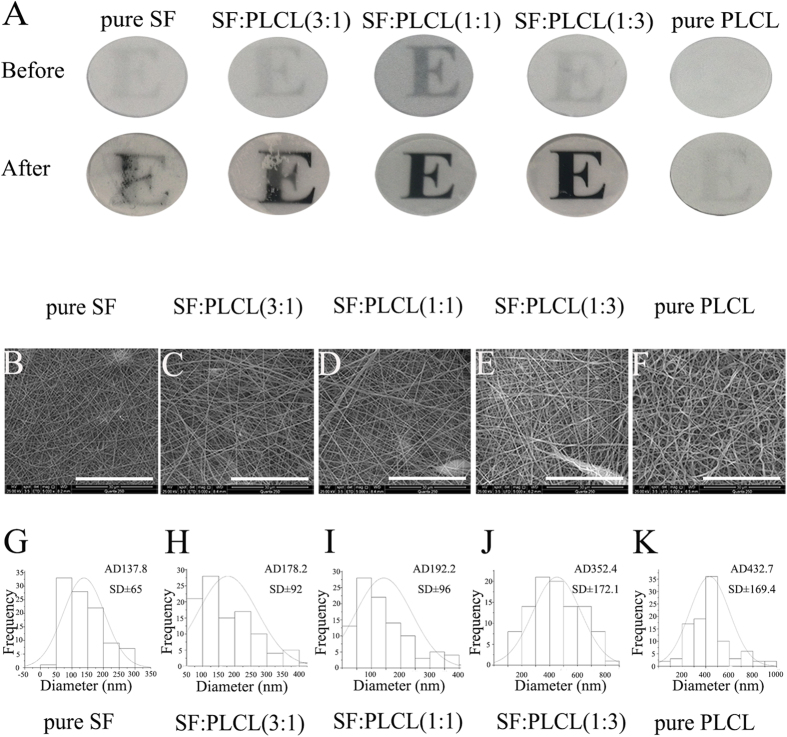
Morphology of electrospun SF/PLCL nanofibrous scaffolds. (**A**): Appearance of electrospun nanofibrous scaffolds before and after immersion in PBS. (**B**–**F**): Scanning electron microscopy images of electrospun nanofibrous scaffolds prepared with different SF/PLCL weight ratios: pure SF, SF:PLCL (3:1), SF:PLCL (1:1), SF:PLCL (1:3) and pure PLCL. Scale bars: 30 μm. (**G**–**K**): Diameter contribution of different SF/PLCL nanofibrous scaffolds. Abbreviations: SF, silk fibroin; PLCL, poly(L-lactic acid-co-ε-caprolactone); PBS, phosphate-buffered saline.

In this study, thin scaffolds (with a thickness of approximately 60–100 um) of pure SF, SF/PLCL blends at different weight ratios, and pure PLCL were successfully produced by electrospinning, and the resultant nanofibrous scaffolds appeared to be homogeneous, as can be observed in Fig. 1A. The nanofibrous scaffolds presented demonstrable differences in transparency before and after immersion in PBS (Fig. 1A), and the blended scaffolds appeared to be more transparent than pure SF or pure PLCL. SEM images depicting the micromorphology of the electrospun nanofibrous scaffolds are shown in Fig. 1B–F, and the average fibre diameter of the scaffolds gradually decreased from 432.7 nm to 137.8 nm with increasing SF content (Fig. 1G–K), demonstrating that all five types of scaffolds were constructed of randomly displayed fibres and completely interconnected pore structures. The equilibrium swelling ratio (ESR) results are shown in Supplementary Fig. S1, and the results showed that the swelling ratio of the scaffolds decreased with an increase in the PLCL content. The swelling ratio of SF:PLCL (1:1) was 0.80 ± 0.53, indicating small changes in the fibre diameter from before to after immersion.

### Mechanical and pore size measurements

**Figure 2:**
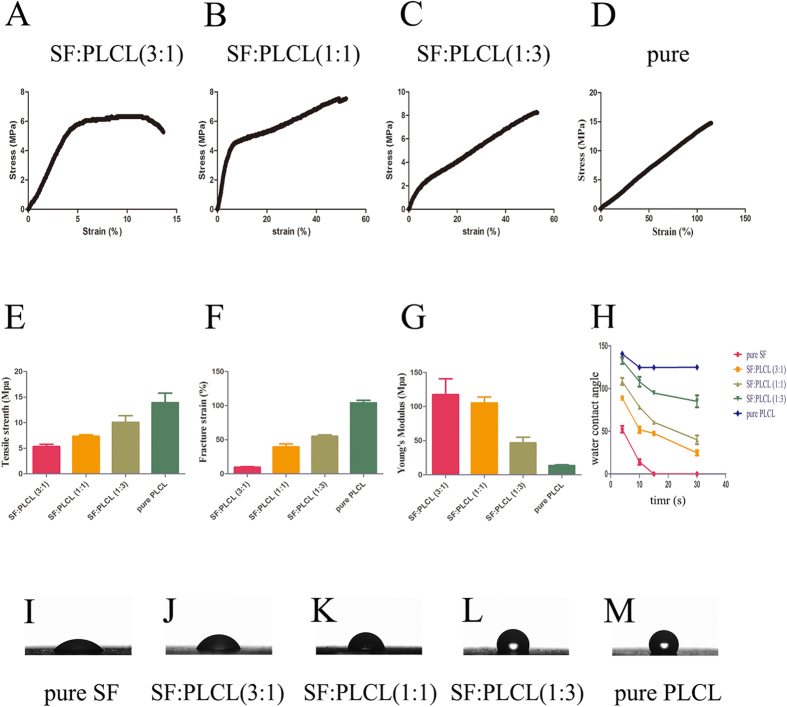
Physical property of different SF/PLCL scaffolds. (**A**–**D**): Tensile stress vs. strain curve of SF:PLCL (3:1), SF:PLCL (1:1), SF:PLCL (1:3) and pure PLCL. (**E**): Tensile strength of SF:PLCL (3:1), SF:PLCL (1:1), SF:PLCL (1:3) and pure PLCL. (**F**): Fracture strain of SF:PLCL (3:1), SF:PLCL (1:1), SF:PLCL (1:3) and pure PLCL. (**G**): Young’s modulus of SF:PLCL (3:1), SF:PLCL (1:1), SF:PLCL (1:3) and pure PLCL. (**H**): The rate of change of the water contact angles obtained for different SF/PLCL weight ratios from 4 s to 30 s. (**I**-**M**): Representative images of the water contact angles obtained for the different SF/PLCL weight ratios at 10 s, showing that the pure SF exhibited the minimum angle.

The mechanical properties of SF:PLCL (3:1), SF:PLCL (1:1), SF:PLCL (1:3) and pure PLCL were reflected by typical tensile stress-strain curves, which are shown in Fig. 2A–D. The quantitative analyses are exhibited in Fig. 2E–G. The pure SF scaffolds were brittle, and thus, their mechanical properties could not be tested. With an increase in the ratio of PLCL, the scaffolds transformed from brittle to flexible, and obvious increases in the average tensile strength and elongation at break were obtained. In addition, the Young’s modulus (SF:PLCL (3:1) 117.62 ± 46.2 MPa vs. SF:PLCL (1:1) 105.34 ± 17.37 MPa vs. SF:PLCL (1:3) 46.95 ± 16.20 MPa vs. pure PLCL 13.562 ± 2.89 MPa) was clearly decreased. The blending of PLCL with SF can markedly improve the mechanical properties of SF to yield employable blended biomaterials. The pore diameter was measured, and the results are shown in Supplementary Figure S2. All the scaffolds were demonstrated to consist of compact pores, with a pore diameter less than 2 μm, indicating that RPCs can grow on the surface of all the scaffolds.

### Water contact angles of different SF/PLCL scaffolds

To clarify the effect of the SF content on the surface properties of the fibrous scaffolds, the wettability was measured through a water contact angle analysis (Fig. 2). Pure SF appeared to be completely hydrophilic, with a water contact angle of 0° at 15 s, whereas pure PLCL exhibited an angle of approximately 120° at 30 s (Fig. 2H). The addition of SF to the surface of the PLCL scaffolds resulted in a smaller water contact angles than those of pure PLCL, which suggested that these scaffolds presented better hydrophilicity in comparison to pure PLCL (Fig. 2H).

### Cell proliferation morphology on electrospun scaffolds

**Figure 3:**
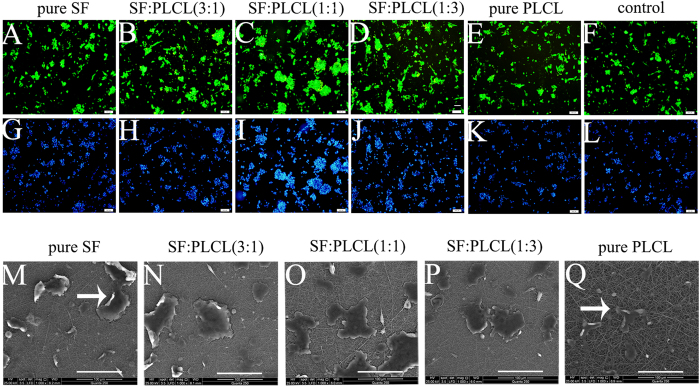
Morphology of RPCs seeded on electrospun SF/PLCL nanofibrous scaffolds. (**A**–**L**): Fluorescent micrographs of GFP+RPCs grown on pure SF, SF:PLCL (3:1), SF:PLCL (1:1), SF:PLCL (1:3) and pure PLCL nanofibrous scaffolds under proliferation conditions for 3 days, and the cell nuclei were counterstained with DAPI. The RPCs cultured on SF:PLCL (1:1) showed the highest cell density. Scale bars: 100 μm. (**M**–**Q**): Scanning electron microscopy images of RPCs grown on pure SF, SF:PLCL (3:1), SF:PLCL (1:1), SF:PLCL (1:3) and pure PLCL nanofibrous scaffolds in proliferation medium for 3 days. Scale bars: 100 μm. Abbreviations: GFP, green fluorescent protein; RPC, retinal progenitor cell.

Figure 3A–F shows fluorescent micrographs of GFP^+^ RPCs on different scaffolds 3 days after their seeding in proliferation medium, and Fig. 3G–L presents the DAPI-stained cell nuclei morphology. In general, the cells showed a healthy morphology on all the scaffolds and appeared in the form of cell clusters, which may indicate that the RPCs maintained an undifferentiated state on the nanofibrous scaffolds. After 3 days of culture, the cell number and diameter of the clusters on all of the blended scaffolds were higher than those found for the control and pure PLCL, and SF:PLCL (1:1) presented the cell clusters with the largest diameter. To visualise the morphology of the RPCs seeded on electrospun scaffolds, SEM images were taken after 3 days in culture (Fig. 3M–Q). RPCs grown on blended SF/PLCL scaffolds adopted a cell-cluster morphology, and the diameters of the cell clusters were larger than those obtained on the pure PLCL scaffolds. In addition, as shown in the SEM micrograph, SF:PLCL (1:1) and SF:PLCL (1:3) scaffolds appear much more attractive for RPC attachment than pure SF or PLCL scaffolds.

### Cytocompatibility detection of SF/PLCL nanofibrous scaffolds on RPC growth

**Figure 4:**
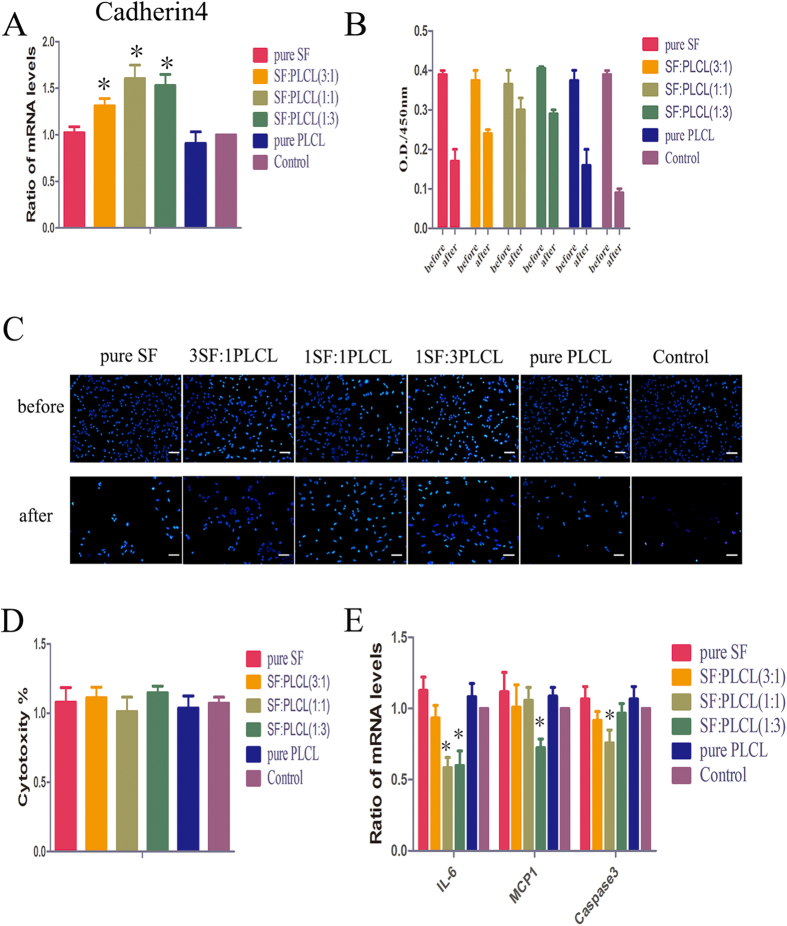
Detection of cytocompatibility of SF/PLCL nanofibrous scaffolds with RPC growth. (**A**): RPCs were cultured on scaffolds for 3 days, and the expression levels of the cell adhesion factor cadherin 4 in the RPC cultures were evaluated, showing that the expression levels of cadherin 4 in the RPC cultures grown on blended SF:PLCL scaffolds were markedly up-regulated compared with the control. (**B**): CCK8 analysis of numbers of RPCs adhered to the substrate surfaces after 12 hours of culture. (**C**): Fluorescence images of DAPI-stained RPC nuclei attached to pure SF, SF:PLCL (3:1), SF:PLCL (1:1), SF:PLCL (1:3) and pure PLCL nanofibrous scaffolds and control glass coverslips before and after three PBS washes. Scale bars: 100 μm (**D**): LDH assays for acute cytotoxicity analysis of SF/PLCL nanofibrous scaffolds with different weight ratios on the health of the cells after 24 h of culture. None of the scaffolds showed obvious cytotoxicity for RPC cultures compared with the control. (**E**): The qPCR results show that in RPCs cultured on electrospun scaffolds for 7 days, the expression levels of the inflammation factors IL-6 and MCP-1 and the apoptotic factor caspase 3 were comparable to or lower than those in the control, demonstrating a marked down-regulation of the expression of IL-6 and caspase 3 in the RPC cultures grown on SF:PLCL (1:1) nanofibrous scaffolds. Notes: The error bars show the standard deviations (n = 3); *P < 0.05. Abbreviations: CCK8, cell counting kit 8; LDH, lactate dehydrogenase; DAPI, 4′, 6-diamidino-2-phenylindole.

To assess cell adhesion, the expression levels of the cell adhesion molecule cadherin 4 were tested. The qPCR results show that the expression levels of cadherin 4 after 3 days in culture were significantly higher in the SF:PLCL (1:1) group than in the control groups, suggesting that SF:PLCL (1:1) scaffolds may be more attractive for RPC adhesion (Fig. 4A). In addition, the attachment of RPCs on the scaffolds after 12 hours was analysed using a CCK8 test. WST-8-formazan (2-(2-4-nitrophenyl methoxy-)3-(4-nitrophenyl)-5-(2,4-disulfonic acid benzene)-2H-tetrazolium monosodium salt formazan, an orange final product in the CCK8 assay) serves as an intermediate to reflect the number of living cells left behind in the cell attachment test. Our results show that the SF:PLCL (1:1) scaffolds had the highest optical density (O.D.), which indicated that a markedly higher number of cells remained on the SF:PLCL (1:1) scaffold than on the other scaffolds (Fig. 4B and Supplementary Fig. S3). DAPI staining of the cells remaining before and after PBS washing further confirmed the above-mentioned results (Fig. 4C). The acute cytotoxicity of nanofibrous scaffolds of different SF/PLCL weight ratios on the health of the cultures after 24 hours was also assessed using the cytosolic enzyme lactate dehydrogenase (LDH) assay, which clearly showed that the SF/PLCL nanofibrous scaffolds exerted no cytotoxicity on RPC cultures (Fig. 4D). In addition, the qPCR results show that RPCs cultured on electrospun membranes for 7 days showed comparable or lower expression levels of the inflammation factors IL-6 and MCP-1 and the apoptotic factor caspase 3 than those in the control group, indicating a marked down-regulation of the expression of IL-6 and caspase 3 in the RPC cultures grown on SF:PLCL (1:1) nanofibrous scaffolds (Fig. 4E). All of these data indicate that all the SF/PLCL scaffolds, particularly the SF:PLCL (1:1) nanofibrous scaffolds, present cytocompatibility for RPC growth.

### Effect of SF/PLCL nanofibrous scaffolds on RPC proliferation

**Figure 5:**
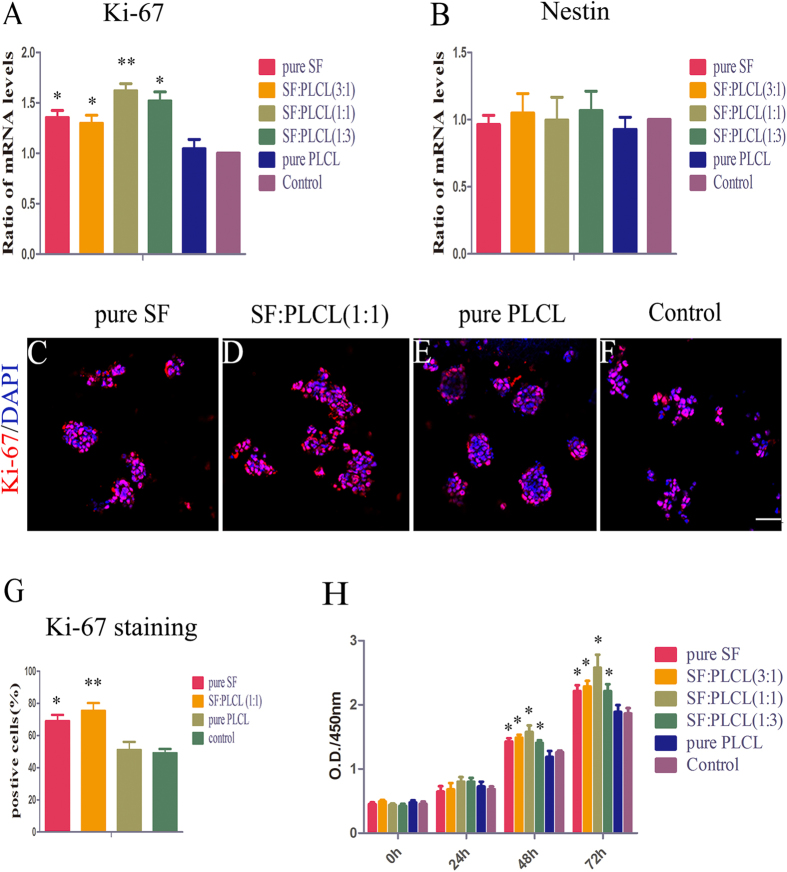
Effect of SF/PLCL nanofibrous scaffolds on RPC proliferation. The expression levels of Ki-67 (a marker of cell proliferation) and nestin (a marker of RPCs) in the RPC cultures grown on the different SF/PLCL scaffolds were evaluated under proliferation conditions. (**A**,**B**): The expression levels of Ki-67 were significantly increased in the RPC cultures grown on the pure SF and blended SF:PLCL scaffolds compared with the other groups. The expression levels of nestin in the cultures grown on all the scaffolds were comparable to those obtained for the control group. (**C**–**F**): After 3 days of culture under proliferation conditions, the RPCs cultured with pure SF, SF:PLCL (1:1), pure PLCL nanofibrous scaffolds and control glass coverslips were immunostained for Ki-67. (**G**): The analysis of the percentage of Ki-67-positive cells in the RPC cultures grown on different scaffolds showed that the percentage was markedly increased in the RPC cultures seeded on SF:PLCL (1:1) compared with the pure PLCL and control groups. (**H**): The CCK8 analysis demonstrated that the RPCs cultured on pure SF and blended SF/PLCL nanofibrous scaffolds showed increased proliferation compared with the other groups, and the cultures grown on SF:PLCL (1:1) had the highest O.D. values. Notes: The error bars show the standard deviations (n = 3); *P < 0.05, **P < 0.01. Scale bars: 100 μm.

To evaluate the effect of nanofibrous scaffolds on RPC proliferation, qPCR was performed (samples were normalised for number of cells), and the results showed that the expression levels of Ki-67, a cellular marker for proliferation, were significantly higher on the pure SF and blended SF/PLCL scaffolds, particularly SF:PLCL (1:1), than in the other groups (Fig. 5A), suggesting that RPCs grown on the SF:PLCL (1:1) nanofibrous scaffolds sustained a more active proliferation state. Nestin expression was indicative of neural stem or progenitor cells and is used as a maker for undifferentiated retinal progenitor cells. Its expression in the cultures grown in the SF:PLCL (1:1) scaffold was comparable to that observed in the control group, indicating that the SF:PLCL (1:1) nanofibrous scaffolds are suitable for RPC self-renewal (Fig. 4B). Immunocytochemistry analysis showed that most cells on the SF:PLCL (1:1) nanofibrous scaffolds stained positively for Ki-67 (75.3 ± 5.77%), and this percentage was markedly higher than that obtained on the pure PLCL scaffolds or the control (glass coverslips) (51 ± 6.67% and 49 ± 3.33%, respectively) (Fig. 4C–G). In addition, the CCK8 analysis, as shown in Fig. 4H, demonstrated that all of the nanofibrous scaffolds were suitable for RPC proliferation. No obvious differences in proliferation capacity were observed in the first 24 h of culture among the different groups. Thereafter, a significantly promoted expansion ability was recorded for the RPC cultures treated with pure SF as well as all those cultured in the blended SF/PLCL nanofibrous scaffolds, which presented O.D. 450 values higher than those obtained for the control group, and the SF:PLCL (1:1) scaffold presented the highest O.D. 450 values. These results indicate that the blended SF/PLCL scaffolds, particularly the SF:PLCL (1:1) scaffolds, could provide a significant advantage as a nanofibrous scaffold material for RPC proliferation, which is important for obtaining a large number of cells for further RPC research as well as for future applications in retinal cell replacement therapy.

### Effects of electrospun SF/PLCL nanofibrous scaffolds on RPC differentiation

**Figure 6:**
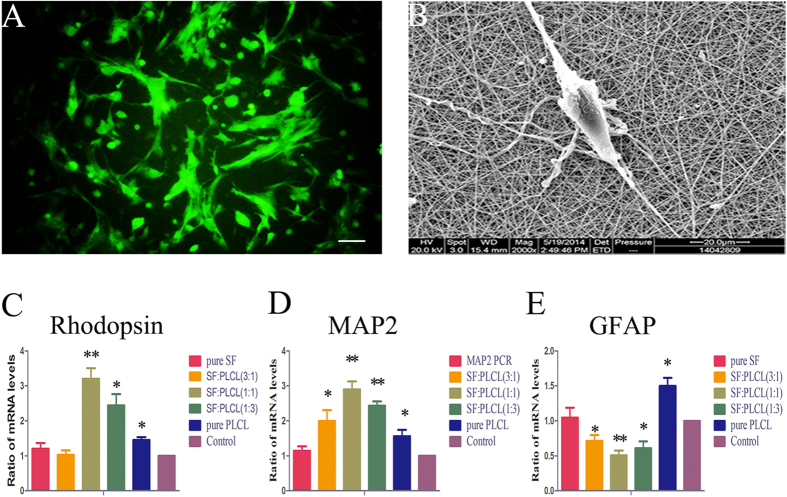
Quantitative evaluation of the effects of the electrospun SF/PLCL nanofibrous scaffolds on RPC differentiation. The cells were cultured under differentiation conditions. (**A**): Fluorescent micrograph of RPC cultures grown on SF:PLCL (1:1). Scale bars: 100 μm. (**B**): Scanning electron micrograph of RPC culture seeded on the SF:PLCL (1:1) nanofibrous scaffold. Scale bars: 20 μm. (**C**–**E**): The expression levels of rhodopsin (a marker of rod photoreceptor cells) and MAP2 (a marker of neuronal cells) were up-regulated 3.1-fold and 2.9-fold, respectively, whereas the expression of GFAP (a glial marker) was markedly down-regulated in the RPC cultures grown on the SF:PLCL (1:1) nanofibrous scaffolds compared with the levels observed in the control group. Notes: The error bars show the standard deviations (n = 3); *P < 0.05, **P < 0.01.

**Figure 7:**
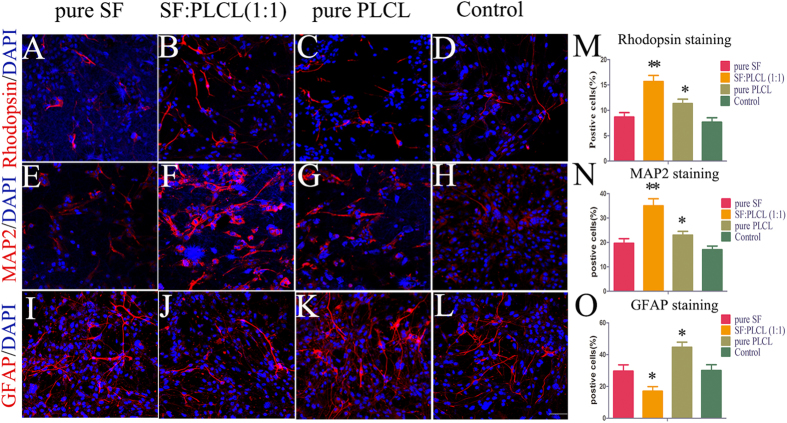
Effects of the SF/PLCL nanofibrous scaffolds on RPC differentiation evaluated by immunocytochemistry analysis. (**A**–**L**): After 7 days of culture under differentiation conditions, the RPCs cultured with the pure SF, SF:PLCL (1:1), pure PLCL nanofibrous scaffolds and control glass coverslips were immunostained for rhodopsin, MAP2 and GFAP, as indicated. (**M**–**O**): The percentages of cells positive for rhodopsin and MAP2 were significantly higher, whereas the percentage of GFAP-positive cells was obviously lower in the RPC cultures grown on the SF:PLCL (1:1) scaffolds than in the other groups. Notes: The bar represents the mean ± standard deviation (n = 3); *P < 0.05, **P < 0.01. Scale bars: 100 μm.

Under differentiation conditions, the cells grown on the SF:PLCL (1:1) scaffolds exhibited normal cell shapes with healthy neurite outgrowth in the fluorescent and SEM micrographs, as shown in Fig. 6A,B. In addition, the effects of SF/PLCL nanofibrous scaffolds on RPC differentiation were also investigated through qPCR and immunocytochemistry. The present study investigated the expression of three key genes involved in retinal development: rhodopsin (a marker for rod photoreceptor cells), MAP2 (a marker for neuronal cells) and glial fibrillary acidic protein (GFAP, a glial marker). The qPCR results, as shown in Fig. 6C–E, show that RPC cultures grown on SF:PLCL (1:1) nanofibrous scaffolds exhibited a marked up-regulation of rhodopsin and MAP2 expression (3.1-fold and 2.9-fold, respectively) in comparison to the control group. By contrast, the expression level of GFAP was markedly lower in the RPC cultures grown on SF:PLCL (1:1) scaffolds than in the control cells. The immunocytochemistry analysis showed that in the RPC cultures grown on the SF:PLCL (1:1) scaffolds, the percentage of cells expressing rhodopsin or MAP2 was significantly higher, whereas the percentage of GFAP-positive cells was clearly lower compared with the other groups (Fig. 7), which is consistent with the qPCR results. These results suggest that RPCs grown on SF:PLCL (1:1) scaffolds under differentiation conditions are more likely to differentiate toward retinal neuronal lineages, including, most interestingly, photoreceptor cells.

Taken together, our data demonstrate that the SF:PLCL (1:1) nanofibrous scaffolds present cytocompatibility for RPC growth. Moreover, the SF:PLCL (1:1) scaffolds can markedly promote RPC proliferation and can robustly accelerate the differentiation of RPCs into retinal neuronal cells, including photoreceptors.

## Discussion

Increasing lines of evidence suggest that the transplantation of RPC with a carrier, instead of the injection of cell suspensions, may be more feasible for RPC survival, differentiation and further migration into an injured retina^17^. An ideal carrier for RPC transplantation should exhibit the following properties: a porous ultrastructure to allow the transport of nutrients and metabolic wastes and good cytocompatibility for cell adhesion, growth and maintenance of multipotency. In the present study, SF and PLCL were chosen for the fabrication of blended scaffolds (SF/PLCL) for two reasons: First, PCL and PLLA have appeared to be advantageous in RPC transplantation experiments^16^,^27^, suggesting that their copolymer PLCL may also perform well. Second, bioactiveSF can provide biological functional groups on the surface of PLCL scaffolds, which may have positive effects on RPC growth, as supported by a previous study that demonstrated that SF scaffolds can support the survival, migration and differentiation of embryonic stem cell-derived neural progenitors^28^; thus, because retinal progenitors are a type of neural progenitor, the above-mentioned lines of evidence indicate that SF may have a similar effect on RPC growth behaviour.

The good mechanical properties of a scaffold are helpful for successful cell transplantation. Retinal tissue has been reported to have an elastic modulus of 0.1 MPa, which indicates that it is a soft and flexible tissue^29^. PLGA has been reported to have an elastic modulus of 1.4-2.8 GPa^30^. In comparison to PLGA, our results showed that SF:PLCL (1:1) has an elastic modulus of 105.3 ± 17.4 MPa, which is closer to that of retinal tissue. However, further study was needed to improve the mechanical properties of the scaffolds in order to better match those of retinal tissue.

In the present study, we show that the SF/PLCL nanofibrous scaffolds exhibited good cytocompatibility, which is a requirement for any biomaterial used for clinical application. Few previous studies have addressed the effects of artificial scaffolds on the expression of inflammation and apoptosis factors by RPCs. In the present study, the expression levels of IL-6, MCP1 and caspase 3 in the RPC cultures grown on the SF/PLCL scaffolds were assessed. IL-6, a pro-inflammatory cytokine, is relevant for intraocular inflammatory diseases^31^. MCP-1 is considered the key gene in the migration of immune-competent cells as well as monocyte infiltration during retinal inflammatory diseases^32^. Caspase 3 is a major terminal cleavage ribozyme in the apoptosis process^33^. In this study, the expression levels of IL-6, MCP-1 and caspase 3 in RPCs cultured on SF:PLCL (1:1) nanofibrous scaffolds were not obviously up-regulated. Moreover, the expression of the cell adhesion molecule cadherin 4 was markedly up-regulated in the cell cultures grown on the SF:PLCL (1:1) scaffolds. All of these data suggest that the electrospun SF:PLCL (1:1) nanofibrous scaffolds present cytocompatibility with RPC growth.

Our data demonstrate that SF:PLCL (1:1) markedly enhanced RPC proliferation, possibly through the following potential mechanisms. First, the resultant nanofibrous scaffolds demonstrate interconnected porous structures with high porosity, as determined by SEM micrography, which would allow an exchange of nutrients and provide a high surface area, thereby mimicking the topographic features of the ECM. A previous study has shown that mimicking the ECM is important for organising cells and for providing signals for cellular responses^34^. In our study, all the scaffolds consisted of compact pores (with a pore diameter less than 2 μm), which would provide larger surface areas for protein adsorption as well as enhance the nanoscale roughness, thus contributing to increases in cell attachment and proliferation^35^,^36^. Second, electro-spinning of SF (with its powerful hydrophilicity) with PLCL will enhance the hydrophilicity of PLCL. The balance between hydrophilicity and hydrophobicity of the scaffold materials may exert positive effects on cell growth^37^. Third, the introduction of biological functional groups, such as -NH_2_ and –COOH, via SF in SF/PLCL may enhance the proliferation of RPCs. Faucheux and colleagues have demonstrated that chemical functional groups, such as -NH_2_ and –COOH, can promote cell-scaffold recognition and subsequent proliferation^38^. In this study, the SF:PLCL (1:1) nanofibrous scaffolds were the most helpful for RPC proliferation, and increasing the amount of SF in the SF/PLCL scaffolds did not yield better effects. A previous study conducted by our group showed that the process of introducing bioactive amino groups on the surface of polymers exerted only a limited positive effect on RPC growth^14^; although the reasons may be various, the strong negative charge on the surface of SF may partly explain this phenomenon^39^.

Inspiringly, our current study demonstrated that SF/PLCL nanofibrous scaffolds, particularly SF:PLCL (1:1) scaffolds, can markedly enhance the differentiation of RPCs toward retinal neurons, including photoreceptors, which are one of the most interesting retinal neuronal cells in retinal cell replacement therapy research. The positive effects of the SF:PLCL (1:1) scaffolds on inducing RPCs to produce more retinal neurons may be attributable to the introduction of bioactive groups, such as -NH_2_, on the surface of PLCL via SF, which may be supported by a previous report that neural stem cells in contact with glass surfaces modified by -NH_2_ were more likely to differentiate into neurons^40^. Furthermore, a previous study demonstrated that a fibre diameter of approximately 200 nm was efficient for neural stem cell differentiation^41^. This may partially explain why SF:PLCL (1:1) (with a fibre diameter of approximately 200 nm) was efficient for RPC differentiation. Although the reasons regarding why SF/PLCL would cause an obvious increase in RPC differentiation towards photoreceptor phenotypes are undiscovered, the possible chemical cues in conjunction with microtopography may have an effect on promoting rod-specific differentiation^15^. The two major clinical subtypes of retinal degeneration (RD), namely retinitis pigmentosa and age-related macular degeneration (AMD), share the hallmark of photoreceptor cell degeneration, which ultimately results in vision loss^1^. The use of SF:PLCL (1:1) scaffolds in this study produced markedly increased populations of photoreceptor cells, which will improve future cell replacement therapies for retinal degenerative diseases.

## Conclusion

In this study, the SF:PLCL nanofibrous membrane was used as a novel scaffold for RPC growth *in vitro*. Our data demonstrate that the SF:PLCL (1:1) nanofibrous scaffolds exhibited cytocompatibility for RPC growth and that these scaffolds can not only markedly promote RPC expansion but also robustly enhance RPC differentiation toward photoreceptors, the most interesting class of retinal neuronal cells for retinal cell replacement therapy. Subsequent investigations will be needed to evaluate the cytocompatibility of SF:PLCL (1:1) nanofibrous scaffolds and to measure the effects of the scaffolds on the proliferation and differentiation of RPCs *in vivo*.

## Materials and Methods

### SF/PLCL nanofibrous scaffolds preparation

Raw silk (Jiaxing Silk, China) was degummed three times with 0.5% (w/w) Na_2_CO_3_ solution at 100 °C for 30 min and then washed with distilled water. The degummed silk was dissolved in a ternary solvent system consisting of CaCl_2_/H_2_O/EtOH solution (1/8/2 in mole ratio) for 1 hour at 70 °C. The SF solution was then subjected to dialysis with a cellulose tubular membrane (250–7u; Sigma Aldrich; USA)^42^,^43^ in distilled water for 3 days at room temperature. After filtration, the SF solution was lyophilised to obtain the regenerated SF sponges. The PLCL copolymer was prepared at a ratio of 50% PLLA to 50% PCL using previously reported methods^44^. For electrospinning, pure SF, SF/PLCL blends with different weight ratios (including SF:PLCL (3:1), SF:PLCL (1:1) and SF:PLCL (1:3)), and pure PLCL were dissolved in hexafluoroisopropanol (HFIP) solvents (Chinese Academy of Sciences, China) to obtain a final concentration of 10% (w/v), and the blending solutions were stirred at room temperature for 6 hours. All of the operations were conducted in a biological safety cabinet (Frontline FHC-1200A, USA). The solutions were then filled into a 2.5-ml plastic syringe with a blunt-ended needle. A syringe with an inner diameter of 0.21 mm was loaded in a syringe pump (789100C, Cole-Parmer, America) and dispensed at a speed of 1.2 ml/h. Using a high-voltage power supply (BGG6-358, BMEICO China), a voltage of 12 kV was applied across the needle and ground collector (a flat grounded steel plate covered with aluminium foil), which was placed at a distance of 12–15 cm, and the nanofibres were collected on the 13-mm glass cover slips on the plate. The resultant scaffolds were fumigated with 75% alcohol for 24 h. They were then rinsed three times with PBS and incubated in proliferation or differentiation medium for 1 hour before the cells were seeded on them. To perform the experiments in the proliferation medium, laminin was coated equally on the substrate of all the groups.

### Characterisation of SF/PLCL nanofibres

The scaffolds were sprayed with Pt by a MP-19020NCTR NeoCoater (JEOL Ltd., Tokyo), and the morphology of the resultant scaffolds was observed with a scanning electron microscope (SEM) (JSM-6701; JEOL, Tokyo, JAPAN) at voltage of 25 kV. The mean fibre diameters were estimated using image analysis software (Image-Pro Plus, Rockville, MD, USA) by selecting 100 fibres randomly observed on the SEM images.

### Equilibrium swelling ratio measurements

Different weight ratios of known scaffold dry weights were immersed in proliferation medium and maintained at 37 °C for 12 h until a swelling equilibrium was reached. The wet scaffolds were then immediately weighed using a microbalance after the excess water lying on the surfaces was absorbed by a filter paper. The equilibrium swelling ratio (ESR) was calculated using the following equation^45^:
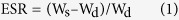
where Ws is the weight of the scaffolds at the equilibrium swelling state and W_d_ is the weight of the scaffolds at the dry state.

### Mechanical measurements

The mechanical properties of pure SF, SF:PLCL (3:1), SF:PLCL (1:1), SF:PLCL (1:3) and pure PLCL were explored by applying a tensile test to all of the specimens prepared from the scaffolds. The mechanical properties were tested using a materials testing machine (H5K-S, Hounsfield, England) at an elongation speed of 10 mm/min. The temperature was controlled at 20 °C, and the relative humidity was maintained at 65%. Each scaffold weight ratio included five samples. The Young's modulus was calculated by measuring the slope of the initial linear region of the stress-strain curves.

### Pore size measurements

The pore sizes were measured using a CFP-1100-AI capillary flow porometer (PMI Porous Materials Int.). The electrospinning nanofibrous membranes were cut into dimensions of 3 cm × 3 cm and were then infiltrated into the wetting liquid with a surface tension of 21st dynes/cm (PMI Porous Materials Int.). As the pressure was increased, the largest hole was the first to open, and the smaller holes then opened. As a result, the pore size distribution of the samples was obtained.

### Water contact angle analysis

Water contact angle measurements were used to explore the surface wettabilities of the electrospun scaffolds. Distilled water was automatically dropped onto the electrospun scaffolds. The images of the droplets on the scaffolds after 10 s were visualised using an image analyser (OCA40, Data Physics, Germany). Furthermore, the angle changes over time were measured automatically. The contact angle was measured three times from different positions, and an average value was calculated.

### Isolation, *in vitro* culture and induction of differentiation of retina progenitor cells (RPCs)

RPCs were separated from the fresh retinal tissue of postnatal-day-1 GFP-transgenic C57BL/6 mice (a gift from Dr Masaru Okabe, University of Osaka, Japan). The cells were cultured in T25 flasks with proliferation medium consisting of advanced DMEM/F12 (Invitrogen, Carlsbad, CA, USA), 1% N2 neural supplement (Invitrogen), 2 mM L-glutamine (Invitrogen), 100 U/ml penicillin-streptomycin (Invitrogen) and 20 ng/ml epidermal growth factor (recombinant human EGF, Invitrogen)^46^. Half of the proliferation medium was changed every 2 days, and the clones were passaged through mechanical isolation or enzymatic trypsinisation at regular 3-day intervals. For RPC differentiation, the cells were trypsinised and seeded at a density of 1 × 10^5^ cells/ml with differentiation medium consisting of 10% foetal bovine serum (FBS) (Invitrogen) without EGF. Half of the culture medium was changed every 2 days. The cells were cultured at 37 °C and 5% CO_2_. All animals were handled according to ARVO animal usage standards, following approval by the animal care and use committee of the Schepens Eye Research Institute, where the original derivation of the cells was performed.

### Morphology of RPCs on the SF/PLCL nanofibrous scaffolds

The morphology of RPCs on the SF/PLCL nanofibrous scaffolds was assessed using a fluorescent microscope (Olympus BX51, Japan) and SEM. The cells were fixed in 4% (w/v) paraformaldehyde (PFA) (Sigma-Aldrich, Poole, UK) in PBS for 15 min at room temperature and then washed three times with PBS. The nuclei were stained with Slow-Fade Gold with DAPI (Invitrogen, Carlsbad, CA, USA), and photographs of GFP^+^ RPCs and DAPI-stained RPCs were taken using a fluorescent microscope. For SEM, the cell-seeded scaffolds were rinsed with PBS and fixed in 4% glutaraldehyde for 2 h at 4 °C. The samples were then dehydrated through a concentration gradient of 30%, 50%, 70%, 80% and 90% ethanol. After air-drying overnight, the samples were sprayed with Pt using a MP-19020NCTR NeoCoater and observed using SEM.

### Cell viability

The effects of pure SF, SF:PLCL (3:1), SF:PLCL (1:1), SF:PLCL (1:3) and pure PLCL on RPC proliferation were assessed using a cell counting kit-8 (CCK-8) (Dojindo, Kumamoto, Japan)^47^. The scaffolds were cut into the same shape as the wells of a 96-well plate using a biopsy punch. The RPCs were trypsinised and suspended in 200 μl of standard proliferation medium/well at a final concentration of 1 × 10^4^ cells/well and cultured in 96-well plates. After 0, 24, 48 and 72 hours, 10 μl of the CCK-8 solution was added to each well. The cells were incubated for another 4 h at 37 °C according to the reagent instructions. The plates were then centrifuged at a speed of 50 g/min for 4 min. Then, 170 μl of supernatant was pipetted into a new 96-well plate. The O.D. value at 450 nm was measured using an ELISA microplate reader (ELX800, BioTeK, USA). The cell viability is expressed as the O.D. 450 value because it is directly proportional to the O.D. value at 450 nm.

### Cell attachment test

The RPCs were cultured in 24-well culture plates for 12 hours; the medium was then replaced with PBS, and the cells were washed three times to remove the un-attached cells. The numbers of cells remaining on both the electrospun scaffolds and the control tissue culture plate surface were detected using the CCK8 method. Briefly, the cells were again incubated with 400 μl of proliferation medium containing 20 μl of CCK8 solution for another 4 hours. The medium was then pipetted into the wells of a 24-well plate and assessed using a microplate spectrophotometer, and the O.D. value at 450 nm for each well was measured. The nuclei were stained with Slow-Fade Gold with DAPI before and after three washes with PBS, and photographs were taken using a fluorescent microscope.

### Cytotoxicity test

To determine the acute cytotoxicity of our scaffolds on RPCs, the amount of the LDH released into the medium was measured using a CytoTox 96 Non-Radioactive Cytotoxicity Assay kit (Promega, USA)^48^. The RPCs were seeded onto scaffolds of different weight ratios for 24 hours. Following the LDH assay kit instructions, briefly, after the culture medium was discarded, 150 μl of the LDH releasing reagent was added, and the plate was incubated for 1 h. Then, 120 μl of liquid was pipetted out to a new 96-well plate, and the LDH testing reagent was added. The O.D. at 450 nm was measured using an ELISA microplate reader (ELX800, BioTeK, USA). The cytotoxicity (%) was expressed based on the value of LDH released into the medium divided by the total LDH activity.

### Total RNA isolation and quality controls

The RPCs were quantified, and the same numbers of RPCs were seeded on the various SF/PLCL scaffolds for 3 days (proliferation condition) or 7 days (differentiation condition). The cell sheets were then treated with 1 ml of TRIzol reagent (Invitrogen) and stirred using a Micro Tissue Grinder (PRO Scientific Inc., Oxford, CT, USA) for 30 s. The total RNA from the cultured cells was extracted according to the manufacturer’s instructions. The samples with OD260/280 nm ratios between 1.8 and 2.0 were used for cDNA synthesis.

### Reverse transcription and quantitative polymerase chain reaction (qPCR)

Using the PrimeScript™ RT reagent kit (Perfect Real Time, TaKaRa, Dalian, China), 1000 ng of total RNA was reverse transcribed into cDNA in a final reaction volume of 20 μl. The produced cDNAs were diluted 10-fold in nuclease-free water (Invitrogen) and used as templates for qPCR. qPCR was conducted in 10 μl solutions containing 5 μl of 2 × SYBR Premix EX Taq™ (TaKaRa), 3 μl of nuclease-free water (Invitrogen), 1 μl of diluted cDNA, and 1 μl of gene-specific primers (Table 1). qPCR was conducted using a 7500 Real-Time PCR Detection System (Applied Biosystems, Foster, CA). The efficiency of the PCR was measured using serial dilutions of the cDNA (1:1, 1:5, 1:25, 1:125, 1:625 and 1:3,125). Each sample was tested in triplicate. The relative mRNA expressions were analysed using the Pfaffl method^49^. Data are expressed as fold change relative to untreated controls, after normalisation to the expression of β-actin.

### Immunocytochemistry

RPCs were cultured on pure SF, SF:PLCL (1:1), pure PLCL and coverslips (VWR, West Chester, PA) coated with laminin (Sigma-Aldrich, Saint Louis, MO) for 3 days or 7 days fixed in 4% PFA in PBS for 15 min at room temperature. The cells were then washed with PBS 3 times and blocked for 1 hour in a blocking solution (PBS containing 10% (v/v) normal goat serum (Invitrogen), 0.3% Triton X-100 (Sigma-Aldrich) and 0.1% NaN_3_ (Sigma-Aldrich))^16^. The samples were then incubated overnight at 4 °C with the following primary antibodies: Ki-67 (mouse monoclonal, BD, 1:200), rhodopsin (mouse monoclonal Millipore, 1:200), MAP2 (rabbit monoclonal, Epitomics, 1:200) and GFAP (mouse monoclonal, Chemicon, 1:200). Then, samples were washed with PBS and incubated with fluorescent secondary antibodies (Alexa Fluor 546 goat anti-mouse or goat anti-rabbit, 1:300 in PBS, BD) for 1 hour at room temperature. The cell nuclei were counterstained with Slow Fade Gold with DAPI (Invitrogen, Carlsbad, CA)^50^. Immunostaining-positive cells were detected using a Zeiss LSM710 laser confocal microscope (Carl Zeiss Microscopy, Oberkochen, Germany). The data were collected using Image-Pro Plus 6.0 (Media Cybernetics, Bethesda, MD), which includes automated counting software. For each RPC culture, 500–1000 cells were counted in random fields.

### Statistical analysis

All the data presented in this paper are shown as the means ± standard deviation (SD) unless otherwise indicated. All the experiments were repeated at least three times. The data from the experimental groups were compared with those from the controls. Statistical analyses of the obtained data were performed by one-way ANOVA, and values of P ≤ 0.05 were considered statistically significant.

## Additional Information

**How to cite this article**: Zhang, D. *et al.* Electrospun SF/PLCL nanofibrous membrane: a potential scaffold for retinal progenitor cell proliferation and differentiation. *Sci. Rep.* **5**, 14326; doi: 10.1038/srep14326 (2015).

## Supplementary Material

Supplementary Information

## Figures and Tables

**Table 1:** Primers used for quantitative qPCR.

Genes	Accession number	Forward (5′-3′)	Reverse (5′-3′)	Annealing Temperature (°C)	Product size (base pairs)
Ki-67	X82786	cagtactcggaatgcagcaa	cagtcttcaggggctctgtc	60	170
nestin	NM_016701	aactggcacctcaagatgt	tcaagggtattaggcaagggg	60	235
Cadherin 4	NM_009867.2	atggttctgctgttcgttgtgt	ggtcgtagtcttggtcctcctc	60	148
IL-6	NM_031168.1	aggagtggctaaggaccaaga	ataacgcactaggtttgccga	60	100
MCP-1	NM_011333.3	acctgctgctactcattcacc	attccttcttggggtcagca	60	148
Casepase-3	NM_004346	catggaagcgaatcaatggact	ctgtaccagaccgagatgtca	60	139
Rhodopsin	NM_145383	tcaccaccaccctctacaca	tgatccaggtgaagaccaca	60	216
MAP2	NM_001039934	agaaaatggaagaaggaatgactg	acatggatcatctggtaccttttt	60	112
β3-tubulin	NM_023279	cgagacctactgcatcgaca	cattgagctgaccagggaat	60	152
GFAP	NM_010277	agaaaaccgcatcaccattc	tcacatcaccacgtccttgt	60	184
β-actin	NM_007393	agccatgtacgtagccatcc	ctctcagctgtggtggtgaa	60	152
