# Supplementary material for: Electrospun SF/PLCL nanofibrous membrane: a potential scaffold for retinal progenitor cell proliferation and differentiation
Source: Sci Rep. 2015 Sep 23;5:14326. doi: 10.1038/srep14326 (PMC4585796; doi:10.1038/srep14326)
Supplement: Supplementary Information [file srep14326-s1.doc]

**Electrospun SF/PLCL nanofibrous membrane: a potential scaffold for retinal progenitor cell proliferation and differentiation**

Dandan Zhang^1^†, Ni Ni^1^†, Junzhao Chen^1^, Qinke Yao^1^, Bingqiao Shen^1^, Yi Zhang^1^, Mengyu Zhu^1^, Zi Wang^1^, Jing Ruan^1^ , Jing Wang^2^, Xiumei Mo^2^, Wodong Shi^1^, Jing Ji^1^*, Xianqun Fan^1^*, Ping Gu^1^*

1. Department of Ophthalmology, Ninth People's Hospital, Shanghai Jiao Tong University School of Medicine, Shanghai, 200011, P.R. China
2. Biomaterials and Tissue Engineering Laboratory, College of Chemistry & Chemical Engineering and Biotechnology, Donghua University, Shanghai, 201620, P. R. China.

Supplementary Figure S1: Equilibrium swelling ratio of SF/PLCL nanofibrous scaffolds.


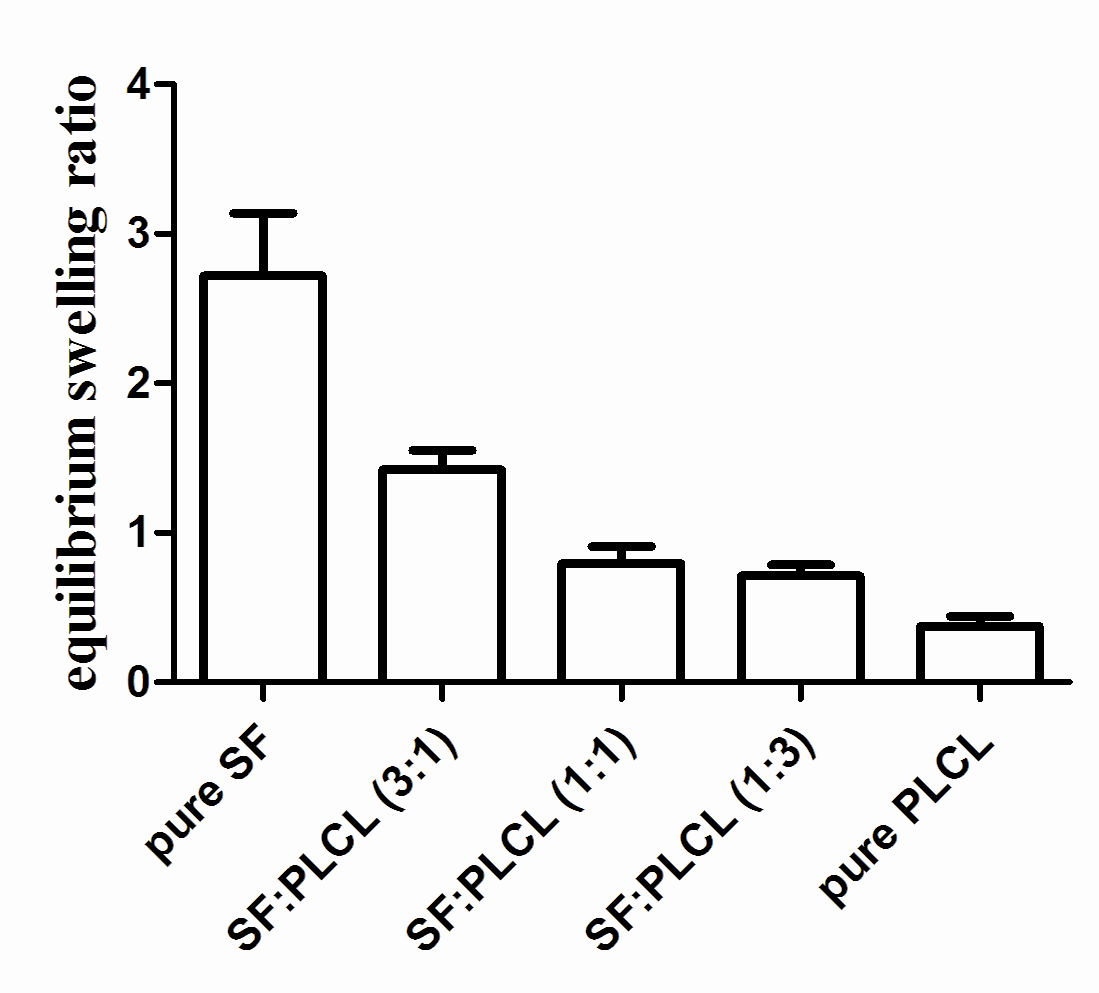


Supplementary Figure S2: Pore size distribution of SF/PLCL scaffolds with different weight ratios.
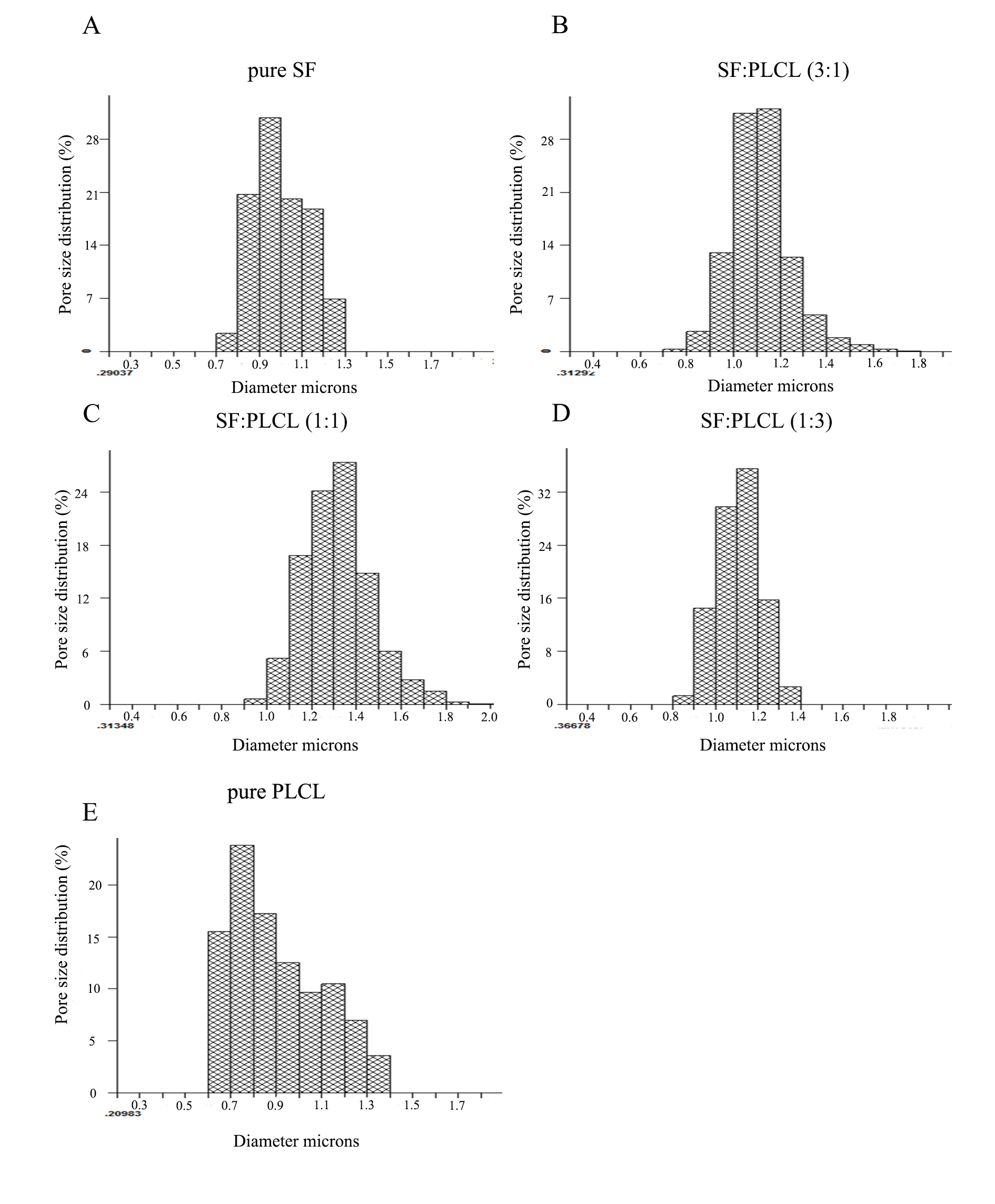


Supplementary Figure S3: Standard curve of CCK8 test on mRPCs.


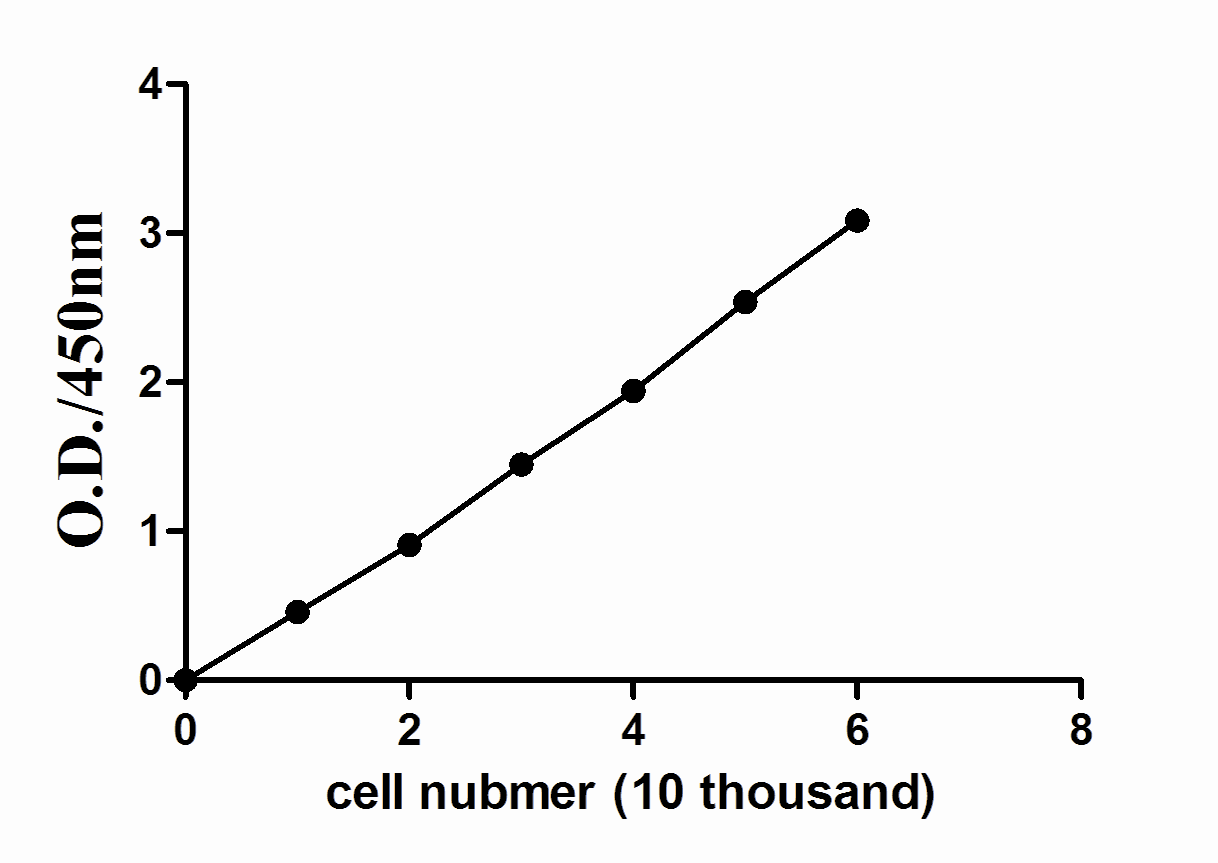


Supplementary Figure Legends

Supplementary Figure S1. Equilibrium swelling ratio of SF/PLCL nanofibrous scaffolds as a function of the volume ratio incubated in proliferation medium at 37°C. The values reported are averages from n = 5 experiments.

Supplementary Figure S2. Pore size distribution of SF/PLCL scaffolds with different weight ratios. All of the scaffolds were found to present structures with compact pores.

Supplementary Figure S3. Standard curve of CCK8 test on mRPCs.
